# N=1-studies In Statin-intolerance; Objectifying Nocebo Effects (NISONE): a study protocol for a randomised controlled trial assessing the implementability of N=1-studies to promote the use of statins

**DOI:** 10.1136/bmjopen-2025-110978

**Published:** 2026-06-25

**Authors:** Ruben Mijnster, Jeanine Roeters van Lennep, Wim Rietdijk, Kübra Akgöl, Marleen Kemper, Daniëlle van den Berg, Hugo van der Kuy, Melvin Lafeber

**Affiliations:** 1 Department of Internal Medicine, Erasmus Medical Center, Rotterdam, Netherlands; 2 Department of Hospital Pharmacy, Erasmus Medical Center, Rotterdam, Netherlands; 3 Apotheek A15, Gorinchem, Netherlands; 4 Department of Internal Medicine, Rijnstate, Arnhem, Netherlands

**Keywords:** Cardiovascular Disease, CLINICAL PHARMACOLOGY, Vascular medicine

## Abstract

**Introduction:**

Lipid-lowering therapies, particularly statins, are central to the prevention of atherosclerotic cardiovascular disease. However, their effectiveness is often compromised by statin-associated muscle symptoms, leading to non-adherence, discontinuation and/or switching to alternative, more expensive therapies such as Proprotein Convertase Subtilisin/Kexin type 9 (PCSK9) inhibitors or bempedoic acid. Recent studies suggest that N=1-interventions may help distinguish true side effects from nocebo-driven symptoms and support reinitiation of statin therapy.

**Methods and analysis:**

The ‘N=1-studies In Statin-intolerance; Objectifying Nocebo Effects’ (NISONE) trial is a study with 249 patients with atherosclerotic cardiovascular disease or familial hypercholesterolaemia who stopped using two or more statins due to perceived symptoms. Participants are randomised (2:1) to an N=1-intervention or usual care. The intervention consists of four double-blind 6-week periods of statin (rosuvastatin 10 mg 1–2 tablets/day or atorvastatin 20 mg 1–2 tablets/day) or placebo treatment during which patients are required to record their symptoms through questionnaires in an application developed for this study. During the subsequent fifth treatment period, feedback on symptoms during the intervention periods is provided in a personalised report, which will be discussed by a healthcare professional. Statin continuation is encouraged if symptoms are similar for statin and placebo periods, but remains voluntary. Statin-intolerant patients in the usual care group will be treated according to the cardiovascular risk management guidelines. The primary outcome, the percentage of patients continuing their statin after 1 year, will be analysed using an odds ratio and its 95% Confidence Interval. Secondary outcomes will be analysed similarly, and cost-effectiveness will be assessed using seemingly unrelated regression equations, adjusted for baseline scores, costs and quality of life.

**Ethics and dissemination:**

The study protocol was approved by a Medical Ethical Research Committee in The Netherlands (EU CT-number 2023-507489-20-00). The study results will be disseminated via peer-reviewed medical journals, conference presentations, advisory boards and, if possible, by using various media channels.

**Trial registration number:**

This trial is registered in the EU Clinical Trials Information System (CTIS) (https://euclinicaltrials.eu/search-for-clinical-trials/?lang=en): 2023-507489-20-00.

**KEY STRENGTHS AND LIMITATIONS**

- The N=1-studies In Statin-intolerance; Objectifying Nocebo Effects trial aims to investigate whether N=1-interventions can sustainably increase statin use in statin-intolerant patients and whether such interventions are feasible in clinical care.
- It will also assess patient-centred outcomes such as medication adherence, satisfaction, quality of life, trust in healthcare providers, as well as cost-effectiveness and acceptance among patients and healthcare providers.
- This trial will provide long-term (4 years) follow-up data, focusing on the effectiveness and feasibility of N=1-interventions to increase statin use in clinical practice.
- The study builds on previous N=1-intervention studies demonstrating the role of the nocebo effect in statin intolerance and their positive impact on statin use.
- Potential recruitment challenges are the possibility of reluctance of patients to participate and the focus of the study on high-risk patients, which may limit generalisability to those at lower cardiovascular risk.

## Introduction

 Lipid-lowering therapies (LLT), particularly statins, are essential in the management and prevention of atherosclerotic cardiovascular disease (ASCVD).^1^,^3^ Statins effectively reduce low-density lipoprotein cholesterol (LDL-C) levels and lower ASCVD risk by 20%–40%.^4 5^ Despite their proven efficacy and safety, 5%–17% of patients report statin-associated muscle symptoms (SAMS) under treatment with multiple different statins, a condition known as statin intolerance, often leading to non-adherence or discontinuation of therapy.^6^ This can compromise cardiovascular outcomes.^7^,^9^ Notably, the reported prevalence of statin intolerance varies depending on study design: double-blind placebo-controlled trials show similar rates for statins and placebo, while observational studies report a higher prevalence.^6 10^ This discrepancy is hypothesised to be partly attributable to the nocebo effect: negative expectations or prior experiences leading patients to misattribute their symptoms to statins.^11^ The National Lipid Association (NLA) defines statin intolerance as the inability to tolerate at least two different statins, with at least one causing any symptoms even at the lowest approved daily dosage.^12^

Recently published studies performed in statin-intolerant patients that included N=1-interventions showed that 85%–95% report no symptom difference between statin and placebo periods, and 50%–65% chose to restart statin therapy after reviewing their personal results.^13 14^ These findings suggest that N=1-interventions can not only clarify symptom attribution but may also support reinitiation of therapy. As such, this approach holds promise for reducing unnecessary discontinuation of statins and minimising reliance on costlier alternatives such as Proprotein Convertase Subtilisin/Kexin type 9 (PCSK9) inhibitors or bempedoic acid.^15 16^ We therefore designed the ‘N=1-studies In Statin-intolerance; Objectifying Nocebo Effects’ (NISONE) trial to evaluate whether implementing an N=1-intervention can promote statin use and assess its feasibility in clinical practice.

### Objectives

The NISONE trial will assess the long-term effects of N=1-interventions on statin use in statin-intolerant patients, extending follow-up to 4 years to evaluate sustained statin reinitiation. Outcomes include not only statin use but also patient-centred measures such as medication adherence, quality of life, satisfaction, medication perceptions, decisional conflict and trust in healthcare professionals. Additionally, the study evaluates the feasibility of implementing N=1-interventions through cost-effectiveness analysis and by assessing both patient and healthcare provider acceptability. If effective, the results will guide the integration of N=1-interventions into routine clinical care.

## Methods

The contents of this study protocol adhered to the Standard Protocol Items: Recommendations for Interventional Trials (SPIRIT) guidelines.^17^

### Study design

**Figure 1:**
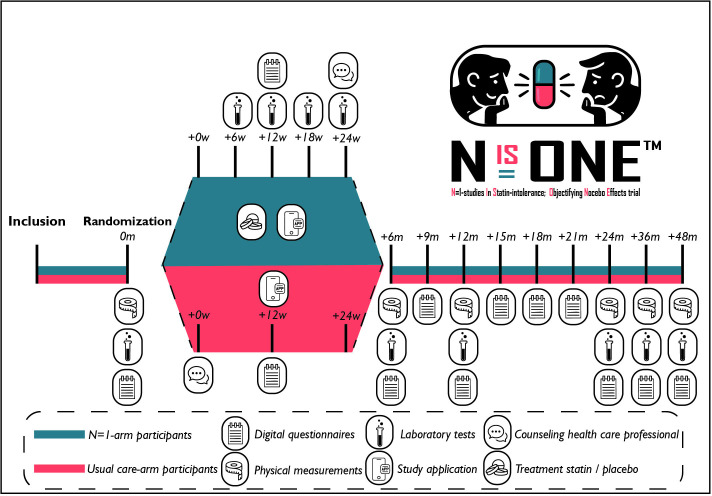
This figure provides an overview of the complete NISONE trial and the outcome measurements that are performed at the different moments during follow-up. m, months; NISONE, N=1-studies In Statin-intolerance; Objectifying Nocebo Effects; w, weeks.

The study comprises a randomised controlled trial (RCT) that is conducted in different (university) hospitals in the Netherlands. The study has two treatment arms in the first half year of the study: an arm in which participants will undergo an N=1-intervention and an arm in which they will receive usual care. The randomisation between these two arms is open-label and participants are randomised among those arms in a 2:1 ratio respectively. A 2:1 randomisation ratio ensures that the majority of participants receive the N=1 intervention, allowing for better-powered analyses for outcomes in which patients identified through the intervention as intolerant due to statin therapy or intolerant due to misattribution and/or nocebo effects are compared with each other. The randomisation will be stratified on study site and the use of ezetimibe at baseline and is performed in the data management system of the study. An overview of this study can be found in figure 1. Inclusions for this study began in April 2025, and we anticipate that the final participant will complete their last visit by December 2032.

Possible treatment sequences for the N=1-intervention are designed in a way to ensure a balanced exposure to statin and placebo treatment across the first four periods of the intervention (ie, two statin periods and two placebo periods). Also, a treatment sequence with two consecutive periods of placebo treatment is excluded to avoid a prolonged use of placebo treatment. Consequently, in the intervention-arm of the study, participants will undergo an N=1-intervention according to three different possible treatment sequences which are double-blinded (table 1). During the N=1-intervention, patients will receive two double-blinded periods of 6 weeks statin treatment (rosuvastatin 10 mg 1–2 tablets/day or atorvastatin 20 mg 1–2 tablets/day) and two periods of 6 weeks placebo treatment. Rosuvastatin and atorvastatin were chosen as the statins of choice due to their relatively high LDL-C lowering potency as compared with other statins. We specifically included the option of either a single daily dose of rosuvastatin 10 mg or atorvastatin 20 mg, or administering a double dose, to allow the treating healthcare professional (HCP) to individualise dosing and thereby optimise the likelihood of achieving guideline-recommended LDL-C targets. The type of statin and dose of the statin that is prescribed to the participant are at the discretion of the treating HCP. During the treatment periods, participants will record their (muscle) symptoms on a Visual Analogue Scale (VAS) at least three times a week. Furthermore, in the sixth week of every treatment period, participants will have their blood drawn for cholesterol levels. Cholesterol levels during these treatment periods are measured to provide participants with insight into their lipid levels under both statin and placebo therapy. This may help motivate patients to continue statin use and also allows verification that the correct treatment was taken during each period. This is important to address potential concerns from participants that treatment periods may have been inadvertently mixed up or that they may have incorrectly taken the assigned medication, which could otherwise lead to doubts about the validity of their individual results. After four periods of 6 weeks in total, participants will be switched to a fifth single-blinded treatment period with the active therapy (statin).

**Table 1:** Treatment sequences in N=1-intervention arm of the study

Treatment sequence	Treatment period				Study results period
	1	2	3	4	
Sequence 1	S	P	S	P	S
Sequence 2	P	S	P	S	S
Sequence 3	P	S	S	P	S

S, statin; P, placebo.

During this fifth treatment period, participants will receive counselling on their individually reported symptoms and cholesterol levels during the treatment periods. They will receive a generated report consisting of several figures and graphs, which will among other things show the number of (muscle) symptoms during statin vs placebo periods, cholesterol levels across the different periods, the distribution of symptoms across different body regions and the extent to which symptoms affected their daily functioning, including any differences between statin and placebo periods. In addition, participants will receive a video that further explains their personal results. Subsequently, they will discuss their individual results in a consultation of approximately 30 min with their treating HCP. During this consultation, the results will be reviewed in detail and further interpretation will be provided, including whether the findings are suggestive of statin-attributable symptoms or symptom misattribution and nocebo effects. A 10 mm higher VAS during statin use is considered as the threshold to attribute the symptoms to statin use.^18 19^ After counselling, statin continuation is advocated if symptoms cannot be attributed to the statin, but it is voluntary and at the discretion of the participant. Participants will be treated according to the clinical guideline and prescribed a PCSK9i if necessary.

In the usual care arm of the study, participants will be treated according to the national cardiovascular risk management (CVRM) guidelines and reimbursement criteria for PCSK9 inhibitors.^20^ When patients with Familial Hypercholesterolaemia (FH) and/or ASCVD report side effects to at least three statins, they are considered statin intolerant as according to Dutch criteria and they qualify for reimbursement of PCSK9i.^21^ At the start of the intervention period, the HCP will discuss the prevalence of SAMS and the strong association with the nocebo effect as this is also stated in the international guidelines on cardiovascular disease prevention.^22^ The HCP will advocate statin use and attempt to rechallenge the patient with a statin or will prescribe another form of LLT, for example, a PCSK9i.

All HCPs who participate in this study will be trained to provide information on the nocebo effect to study participants. In this training, HCPs will receive practical tips focusing on communication about the nocebo effect. This training will be provided by the study coordinator (RM) and two dedicated behavioural psychologists involved in this project, one of which was also a patient advocate that was involved in the study.

### Study treatment

In the treatment arm of the N=1-intervention, participants will be administered tablets of rosuvastatin 10 mg 1–2 tablets/day or atorvastatin 20 mg 1–2 tablets/day along with their corresponding placebo tablets. These generic tablets have been developed and manufactured by Apotheek A15 (Gorinchem, The Netherlands) under a Good Manufacturing Practice license and the medication will be supplied to us. We will conduct two separate studies to demonstrate bioequivalence of atorvastatin and rosuvastatin with the marketed products (Lipitor and Crestor respectively) as comparator.

### Study population

Our study population will consist out of adult patients at high risk of ASCVD based on having FH or at very high risk based on having a diagnosis of previous ASCVD. All patients eligible for inclusion reported statin-related side effects while on ≥2 statins. The study will be conducted in at least 11 hospitals in The Netherlands, of which the academic medical centre of the Erasmus MC is the coordinating centre. Exclusion criteria for this study are:

- a contra-indication for statin therapy;
- any condition that causes severe chronic muscle pain (ie, myopathy or myositis);
- a history of severe mental illness;
- a history of liver cirrhosis or severe renal insufficiency;
- an ASCVD event in the past 3 months;
- an inability or insufficiency to fill in the digital questionnaires;
- a clinical judgement of the HCP that LLT cannot be withheld in a patient with a recent ASCVD event in the past 12 months;
- the clinical judgement of the HCP that LLT is no longer relevant for the prognosis of the patient;
- the clinical judgement of the HCP that participation in the study is unethical or unwanted.

### Recruitment of patients

Patients will be recruited via the outpatient clinics of all participating centres in this study. Recruitment will take place through cardiology and vascular medicine clinics in regional hospitals and academic medical centres, as these specialists are the only healthcare professionals authorised to prescribe PCSK9i to adults in the Netherlands. Potential participants will be asked whether they are interested in participating in the trial after receiving information about the study from their treating healthcare professional. They will be given at least 1 week to consider whether or not they wish to participate. With the consent of potential participants, reasons for non-participation will be recorded in order to assess both the proportion of eligible patients who agree to participate and the reasons for declining participation.

### Measurements and data collection

An overview of the study assessments at different moments during follow-up can be found in figure 1. During the study, participants will receive follow-up by means of clinic visits and by means of digital surveys. During study visits, HCPs will measure blood pressure, heart rate, weight, waist circumference and physical activity. They will also record lifestyle habits and record all medication dispensing data of the pharmacy and (changes in) medical history. HCPs will record their acceptability of the intervention using a questionnaire at 6, 12 and 24 months after randomisation.

During the 4 years of follow-up, participants will be asked to complete a survey on multiple timepoints of the study (figure 1). All items that these questionnaires consist of are:

- demographics;
- self-reported medication use;
- barriers to medication adherence (open-ended question);
- reasons for stopping medication (open-ended question);
- changes in lifestyle (open-ended question);
- participant acceptability of N=1-intervention (self-made, non-validated questionnaire with statements about acceptability on a 5-point Likert-scale; disagree-agree);
- 5-item Medication Adherence Reporting Scale (MARS-5);
- EuroQoL-5 Dimensions-5 Levels (EQ-5D-5L);
- Set of Brief Screening Questions (SBSQ);
- Beliefs about Medicines Questionnaire (BMQ);
- Patient Experiences and Satisfaction with Medication (PESaM);
- Decisional Conflict Scale (DCS);
- Wake Forest Physician Trust Scale (WFPTS);
- Psychological Inventory of Financial Scarcity on 4 items (PIFS-4);
- iMTA Medical Consumption Questionnaire (iMCQ);
- iMTA Productivity Costs Questionnaire (iPCQ).

### Planned outcomes

The primary outcome of the study is the incidence of self-reported statin use at 12 months after randomisation, which is a binary outcome measure (yes/no). We specifically chose this outcome as the primary endpoint because the main objective of the study is to determine whether N=1-interventions, compared with usual care, can increase statin use in patients in routine clinical practice. We considered the 12-month timepoint to be the most appropriate to evaluate this outcome, as we believe this timepoint, approximately 6 months after completion of the N=1-intervention, best reflects patients’ willingness to continue statin therapy in the longer term, beyond the immediate influence of the intervention. Secondary outcomes of this study are focused on use of cholesterol-lowering medication at different timepoints, cholesterol levels at different timepoints, cost-effectiveness, satisfaction with medication and multiple outcomes that assess the applicability of N=1-interventions in clinical care. All secondary outcomes that are studied are:

- statin use;
- PCSK9i use;
- LDL-C levels;
- quality of life (EQ-5D-5L questionnaire);
- reported symptoms;
- cost-effectiveness (iMCQ and iPCQ questionnaire, among others);
- views on medication (BMQ questionnaire);
- satisfaction with statins (PESaM questionnaire);
- decisional conflict (DCS questionnaire);
- trust in the HCP (WFPTS questionnaire);
- HCP satisfaction with intervention (self-made, non-validated questionnaire with statements about acceptability on a 5-point Likert-scale; disagree-agree);
- treatment concordance during intervention;
- physical and mental complaints during intervention.

### Sample size calculation

A power analysis was performed to estimate the required number of participants for the study. This calculation assumed that 30% of patients will continue using statins after the N=1-intervention compared with 12% in the usual care group and included a drop-out margin of 25%. We have based the assumption that 30% of patients will continue statin therapy in the N=1-intervention arm of the study on four previous N=1-studies conducted in patients with statin intolerance that assessed the proportion of patients who restarted or were willing to restart statin therapy.^13 14 23 24^ Two of these studies reported the lowest lower bounds of the 95% Confidence Intervals (95% CIs) for this outcome of 29%.^23 24^ To ensure a conservative estimate for our power calculation, we therefore based our assumption on this lower bound. We have also assumed that 12% of patients in the usual care arm will continue statin therapy, which we consider a cautious estimate. However, we anticipate that some contamination bias may occur, as physicians may apply insights gained from the N=1-intervention arm in routine care, for example, through improved communication regarding statin intolerance and nocebo effects. This could lead to a higher proportion of patients in the usual care arm choosing to continue treatment.

Based on a two-sided alpha level of 0.05 and 80% power, our calculation showed that a total of 247 participants are needed for the study that has a 2:1 ratio of participants in the intervention and control arms respectively. Our trial has been funded for 249 participants, so we will include this amount of participants.

### Data analysis plan

For our primary endpoint, the percentage of patients that continue their statin will be analysed by calculating an odds ratio that compares the participants randomised in the intervention and usual care arm and by calculating its associated 95% CI. Secondary endpoints will be analysed by applying the same strategy as for the primary endpoint with regard to categorical variables. For continuous variables, differences will be reported with a mean (SD) or median (IQR) depending on the distribution of the data and differences will be tested using a t-test or Mann-Whitney U test. For the cost-effectiveness analysis, an empirical cost-effectiveness analysis (CEA) and cost-utility analysis (CUA) will be conducted, which will employ seemingly unrelated regression equations (SURE), adjusted for baseline scores, baseline costs and quality of life.

In the case of missing baseline data, data will be imputed on the basis of other baseline characteristics like age, sex, Body Mass Index (BMI) and history of ASCVD. As convention, the outcome and follow-up values are not imputed. Spurious data will be cleaned from the dataset.

### Monitoring and safety

Monitoring of the study will be conducted according to the national regulations for the monitoring of clinical trials. Every study site will be visited for a monitor visit at least one time a year. At least 10% of the data will be verified per monitoring visit at a specific study site.

Rosuvastatin is used for many years in usual care and has a well-documented safety profile. On the basis the trial treatment is clinically indicated for their medical condition and the well-known safety profile of the trial treatment, we do not register adverse events (AEs).

Serious adverse events (SAEs) and suspected unexpected serious adverse reactions (SUSARs) will be registered if self-reported by the participant or reported by the healthcare provider. To minimise the risk of unreported events, at all study visits, the participant will be actively asked for SAEs and SUSARs.

### Patient and public involvement

Three patients that suffered from cardiovascular disease formed a patient panel for this study. The patient panel was actively involved in the study and played a key role in its development. They provided feedback on the design of this study and the writing of the study protocol from the patient perspective. They also contributed to the creation of all materials to be sent to patients, such as the questionnaires in the N=1-intervention and the reports patients would receive. Regular meetings were held with the entire study team and participating centres, in which the patient panel also took part.

## Ethics and dissemination

The study will be conducted according to the principles of the Declaration of Helsinki and informed consent of participants will be obtained prior to start of the study.^25^ An example of the participant information leaflet together with the informed consent form is provided in online supplemental file 1. The study protocol was approved by a Medical Ethical Research Committee in The Netherlands (EU CT-number 2023-507489-20-00). The study results will be disseminated via peer-reviewed medical journals, conference presentations, advisory boards and, if possible, by using various media channels.

## Supplementary material

10.1136/bmjopen-2025-110978 online supplemental file 1
