# Supplementary material for: N=1-studies In Statin-intolerance; Objectifying Nocebo Effects (NISONE): a study protocol for a randomised controlled trial assessing the implementability of N=1-studies to promote the use of statins
Source: BMJ Open. 2026 Jun 25;16(6):e110978. doi: 10.1136/bmjopen-2025-110978 (PMC13311702; doi:10.1136/bmjopen-2025-110978)
Supplement: online supplemental file 1 [file bmjopen-16-6-s001.docx]

**Subject information for participation
in medical research**

**Side effects of statins due to negative expectations of the drug**

*N=1-studies In Statin-intolerance; Objectifying Nocebo Effects (NISONE) study*

**Introduction**

Dear Sir/Madam,

With this information letter we want to ask you if you want to participate in medical-scientific research. Participation is voluntary. You are receiving this letter because you have had side effects with medicines that are needed to lower your cholesterol, so-called statins. Here you can read what kind of research it is, what it means for you, and what the advantages and disadvantages are. It's a lot of information. Would you like to read through the information and decide whether you want to participate? If you would like to participate, please fill in the form provided in Annex E.

**Ask your questions**

You can make your decision with the information provided in this information letter. In addition, you can also:

- Ask questions to the researcher who gives you this information.
- Talk to your partner, family or friends about this research.
- Ask questions to an independent expert. In this study, this is Dr. Margreet Wagenmakers, internist-endocrinologist. For contact details see Appendix A.
- Read the information at: [www.rijksoverheid.nl/mensenonderzoek](http://www.rijksoverheid.nl/mensenonderzoek).

1. **General information**

Erasmus MC has set up this study. Below, we always refer to Erasmus MC as 'the client'. Researchers, who can also be treating doctors, nurses and pharmacists, are conducting the study in 7 different hospitals in the Netherlands. This research was paid for with money from a grant from the Dutch government (ZonMw).

Participants in a medical-scientific study are often referred to as test subjects. Both patients and people who are healthy can be test subjects.

This study requires 249 test subjects. The medical ethics review committee MREC Amsterdam UMC has approved this study.

**2. What is the purpose of the study?**

In this study, we look at whether the side effects in subjects taking statins are caused by the drug itself or are related to the negative expectation that people have when using drugs. The development of complaints with a negative expectation is called the 'nocebo effect'. We are investigating whether it is useful to have test subjects alternate between using a real statin tablet and tablets without an active ingredient in it (placebo) for periods of six months. Previous research has shown that the vast majority of patients experience the same number of side effects with a statin as with placebo. This study can help to make a wise decision to stop or continue using the statin in the long term. If you manage to continue with a statin, it is often not necessary to use a new, sometimes much more expensive cholesterol-lowering drug.

1. **What is the background to the study?**

Cholesterol-lowering drugs, so-called statins, are very effective in lowering the risk of cardiovascular disease. In addition, statins are also very safe to use. However, statins suffer from a reputation problem. In scientific research, side effects are seen in only 5% of the test subjects when using statins. In the consulting room, however, up to 30% of patients who use statins report complaints such as muscle pain. A significant proportion of patients eventually stop taking the statin. Previous scientific research shows that more than 90% of these patients are likely to develop the side effect of muscle pain not so much because of the active ingredient in the statin tablet. The cause of the symptoms may be the expectations of the side effects you can get from using a statin: the so-called 'nocebo effect'.

Lowering cholesterol is important to minimize the risk of cardiovascular disease. If patients cannot tolerate statins, other medications are available that can lower cholesterol in the blood. These drugs are called PCSK9 inhibitors. PCSK9 inhibitors are just as effective as statins, but are thousands of dollars more expensive to use per year. Because these drugs are more than 250 times more expensive (but not better or more effective) than statins, these drugs have a major effect on healthcare costs in the Netherlands. If patients can tolerate a statin, the use of a PCSK9 inhibitor is often not necessary.

With this study, we would like to see if we can help patients make a wise choice in the further use of a statin. We want to investigate whether it helps in the choice to have subjects alternate between taking both real statin tablets and placebo tablets over a longer period of time.

1. **How does the study work?**

*How long does the study take?*

Will you participate in the study? Then that takes about 4 years in total. The first 6 months are the most intensive.

*Step 1: are you suitable to participate?*

We first want to know if you are suitable to participate. That is why the researcher conducts a number of tests:

- Physical. For example, the researcher measures your blood pressure, heart rate, height, weight and waist circumference.
- Blood test. To do this, the examiner will take some blood from you. We test your blood for cholesterol levels, blood sugar and abnormalities in the salts in the blood. We will tell you if we notice any deviations in these values.
- Examination of your medical history, previously reported side effects on cholesterol-lowering medication, and further medication use.

*Step 2: the study itself*

For this study, we make 2 groups, for which the course of the study in the first half of the year is different:

- Group 1. In this group, you will receive 5 treatments of 6 weeks in the first six months of the study. During these 6-week treatment periods, you will be given a real statin tablet (rosuvastatin) or tablet without an active ingredient (placebo). So these are 2 or 3 periods with a statin tablet and 2 or 3 periods with a placebo tablet. You do not know which treatment you will receive and when. In the first 4 periods of 6 weeks, you keep track of physical and psychological complaints via questionnaires in an Erasmus MC telephone app (DigiZorg application). You may keep track of the complaints daily, but in any case you will be asked to fill in the data 3 times a week. The last week of the 6 weeks the complaints must be filled in daily. After a period of 6 weeks, you will switch treatment and continue with the next treatment period. During treatment period 5, you will be informed of your results on the basis of the questionnaires you have completed. This will be followed by a conversation with your attending physician or nurse specialist. The practitioner will discuss whether there is a difference in symptoms when using the statin tablet or placebo. You can then decide for yourself whether you want to continue with the statin or stop treatment. If necessary, another cholesterol-lowering medication can be prescribed for you.
- Group 2. In this group, you will receive information from your treating physician or nurse specialist about statins, the side effects of statins and the possible explanation of these side effects. The practitioner will then discuss whether it is necessary to try another statin before switching to another cholesterol-lowering drug. In this group, too, you keep track of your symptoms by means of questionnaires in a telephone app of the Erasmus MC (DigiZorg application). You don't have to do this as often in this group as in group 1, namely only once a week. In this group, you will also not gain insight into your personal results of complaints after six months and will not discuss these results with your attending physician or nurse specialist.

A draw determines whether you end up in group 1 or group 2. The chance that you will end up in group 1 is equal to 67% (2 of 3) and the chance that you will end up in group 2 is equal to 33% (1 of 3). You and the practitioner will know which group you are in, but not in which period you will receive a statin tablet or placebo tablet if you are assigned to group 1. The treatments will be announced to you during the conversation with your attending physician or nurse specialist. If it is important for your health, the treatment used can be sought earlier.

For this study, it is necessary that you are registered as a patient at Erasmus MC, even if you are not being treated here. We send the medicines from Erasmus MC and the DigiZorg phone app is only available if you are registered at Erasmus MC.

*Step 3: Surveys and measurements*

Depending on which group you end up in in this study, we ask you to come to the hospital a number of times for examinations and conversations with your practitioner. We ask participants in both groups to keep track of their side effects in the first six months (4 times 6 weeks) via questionnaires in the DigiZorg application on the Erasmus MC mobile phone. If you are assigned to group 1, you will keep track of your symptoms at least 3 times a week and 1 time a day in the last week of a period. If you are assigned to group 2, you will keep track of your symptoms once a week in the first six months. For the study, it is necessary that you come to the hospital a maximum of 8 times in the first year. After that, we ask you to come to the hospital once a year for the remaining 3.5 years of the study. A visit takes about half an hour to an hour.

We do the following tests:

- Physical. The researcher will measure your blood pressure, heart rate, height, weight, and waist circumference.
- Examination of your blood. To do this, the researcher takes 1-2 tubes of blood at a time. During the entire study, we will take about 60-100ml of blood from you, about 10ml at a time. The total amount of blood we collect from you depends on the group in which you are assigned in the study and the number of tubes of blood that are taken. This amount does not cause any problems in adults. In comparison, someone who donates blood at the blood bank gives 500 ml of blood at a time. In the blood test, we look at your cholesterol levels, blood sugar and signs of muscle damage in your blood.

Appendix C states which actions and measurements we take at each visit.

We also collect data about your ethnicity as this may be relevant to our research. Previous research has shown that the extent to which people experience side effects from statins can be both biologically and culturally determined. By registering your ethnicity, we can take this factor into account in our research.

You will fill in a total of 11 different questionnaires during the study. These are not the same questionnaires that you have to fill in in the DigiZorg phone app. These questionnaires include your quality of life, your satisfaction with the medication, taking your medication and whether you have had contact with other healthcare providers. Completing these questionnaires is digital via computer or telephone and takes about half an hour at a time. In these questionnaires, we also ask you about your ethnicity. This is important for our research, because we suspect that side effects of statins could be culturally determined. By asking for your ethnicity, we can take this into account when we process the results of the survey.

*What is different from regular care?*

We ask all participants to keep track of their symptoms in the first half of the year via questionnaires that are built into Erasmus MC's DigiZorg phone app. Participants in group 1 will have to come to the hospital a total of 4 extra times for blood sampling in the first six months of the study. After this six months, there are in principle no more appointments than during usual care. During the rest of the study, we will ask participants to complete online questionnaires, donate blood and come to the hospital for check-ups. These checks would also take place if you did not participate in the study.

1. **What agreements do we make with you?**

We want the research to go well. That is why we make the following agreements with you:

- You take the statin tablet or the tablet without an active substance in it (placebo) in the way explained to you by the investigator.
- During this study, you will not participate in any other medical-scientific study.
- You come to every appointment.
- You carry the participant card of the study with you, for example in your wallet. It states that you are participating in this study. And who to warn in the event of an emergency. Show this card when you visit another doctor.
- You should contact the researcher in these situations:
  - You want to start taking other medications. In the first instance, contact your own pharmacy to ask whether this is possible in combination with the prescribed statins as part of the study.
  - You will be admitted to hospital or treated.
  - You suddenly start having problems with your health.
  - You no longer want to participate in the study.
  - Your phone number, address, or email address changes.

*Can you or your partner become pregnant during the study?*

Women who are pregnant or breastfeeding cannot participate in this study. Women must also not become pregnant in the first 12 months of the study. It is therefore important for women to use good contraception in the first 12 months of this study or that their partner uses a contraceptive. For example, you can think of the following forms of contraception:

- A condom.
- The contraceptive pill.
- A contraceptive patch.
- A vaginal ring with hormonal contraception.
- The contraceptive injection
- An implantation implant (Implanon)
- A hormonal or copper IUD

This study can have consequences for an unborn child if statin is used during pregnancy. It is unclear whether statins during pregnancy can cause birth defects to the baby. Nevertheless, we go for optimal safety and therefore avoid taking a statin during pregnancy. The researcher will tell you how best to prevent pregnancy. Talk to your partner about this.

*Still pregnant?*

Do you get pregnant during the study? Then let the researcher know immediately. In consultation with the investigator, you must then stop the medication as soon as possible and possibly with this study.

1. **What side effects, adverse effects or discomforts can you experience?**

Statins are safe drugs, but can cause side effects. Note: immediately notify the researcher if you experience the following:

- Very severe muscle pain
- Very severe muscle weakness
- Severe skin abnormalities
- Complaints consistent with allergies such as shortness of breath, wheezing and rapidly occurring skin abnormalities

The following side effects occur in up to 10% of users:

- Muscle pain, it can help to take a painkiller such as paracetamol.
- Feeling of weakness
- Abdominal complaints, it can help to take a painkiller such as paracetamol.
- Nausea
- Decreased bowel movements/constipation
- Vertigo
- Headache, it can help to take a painkiller such as paracetamol.

The following side effects are rare, but can be serious:

- Allergic reaction
- Inflammation of the liver
- An inflammation of the pancreas
- Low values of platelets

Among the complaints mentioned above, it should be mentioned that statins have been used for many years. The safety of statins has been extensively researched and demonstrated. Therefore, the use of statins is safe. More information about the statin we are using in this study (rosuvastatin) can be found in the package leaflet in Appendix D. Are you participating in the study? Then you will receive the package leaflet with the medicine.

*What are the possible inconveniences of measurements during the study?*

This study entails little to no physical discomfort. Only the blood draws may hurt a little or you may get a bruise at the place of the body where the blood is taken due to the blood draws.

1. **What are the advantages and disadvantages of participating in the study?**

Participating in the study can have advantages and disadvantages. We list them below. Think about this carefully, and talk about it with others.

In principle, participating in this scientific study has no benefits for the participant. The research was set up to gain more scientific knowledge. This study may help to make a wise choice to stop or continue using the statin. In group 1 of our study, you can see if you experience more muscle pain when you take a statin tablet compared to when you use a placebo tablet. We expect that participants who experience the same number of side effects of muscle pain with a real statin tablet as with a tablet without an active ingredient will be more likely to continue using a statin.

Participating in the study can have these disadvantages:

- You may experience the side effects of statins again.
- If you are assigned to Group 1, you will not receive cholesterol-lowering medications for 2 or 3 periods of 6 weeks, which may cause your cholesterol levels to rise during this time. There are also some studies that show an increased risk of cardiovascular disease when you are not taking a statin, if you have recently had cardiovascular disease. However, the evidence for this is very limited, and many other studies do not show this effect. In general, statins are only considered to have a beneficial effect on cardiovascular disease if they are used for at least 2 to 3 years. Therefore, the disadvantage of not using statins for a short period of time is minimal. For the long term, it is of course important to use a statin or other cholesterol-lowering drug.
- If you have previously used rosuvastatin and experienced side effects, there is a chance that you will notice more side effects during (part of) the study due to this negative experience.
- You may be bothered by the blood draws during the study.
- Participating in the study will cost you extra time.
- You must adhere to the agreements associated with the study.

*Don't want to participate?*

You decide whether to participate in the study. Don't want to participate? Then nothing will change and your doctor or nurse specialist will continue to treat you as necessary. Your doctor can tell you more about the treatment options available.

1. **When does the study stop?**

The researcher will let you know if there is any new information about the study that is important to you. The researcher will then ask you whether you will continue to participate.

In these situations, the study stops for you:

- If the end of the entire study is reached after 4 years.
- If you became pregnant during the first year of enrolment in the study.
- If you want to stop the study yourself. You can do so at any time. Then report this to the researcher immediately. You don't have to tell why you are quitting. You will then receive the normal treatment for patients who do not tolerate statins well due to the side effects.
- If the researcher or your practitioner thinks it is better for you to stop. The investigator will still invite you for a follow-up check.
- If during the interim results of this scientific study it appears that there is little effect of the various treatments.
- If one of the following agencies decides that the investigation should stop:
  - Erasmus MC
  - the government, or
  - the medical ethics committee that assesses the research.

*What happens if you stop the study?*

The researchers will use the data and body material such as the blood you have given during the blood draws collected up to the time of stopping. If you wish, collected body material can be destroyed. Please inform the researcher.

The entire scientific research is over when all participants are finished.

1. **What happens after the study?**

*Can you continue to take the medication?*

If you have any leftover medication after the study, they must be returned to the hospital after treatment. Your practitioner can prescribe the same statin or other medications so that you can pick it up at the local pharmacy.

*Will you receive the results of the study?*

If you end up in group 1 of the study, you will be informed of all your first individual results of the study six months after the study has started. After the study has been completed in its entirety, the investigator will inform all participants of the most important general outcomes of the total study.

1. **What do we do with your data and body material?**

Will you participate in the study? Then you also give permission for your data and body material (blood samples) to be collected, used and stored.

*What data do we keep?*

We store this data:

Your name

Your gender

Your address

your date of birth

data about your health

(medical) data that we collect during the study

*What body material do we store?*

We collect, use and store tubes of blood that we have collected from you.

*Why do we collect, use and store your data and body material?*

We collect, use and store your data and your bodily material in order to be able to answer the questions of this study and to publish the results. Data and/or body material can be used by the client.

*How do we protect your privacy?*

To protect your privacy, we provide a code for your data and your body material. We only put this code on all your data and body material. We keep the key to the code in a secure place with the researcher. When we process your data and body material, we only use that code. Even in reports and publications about the investigation, no one can recall that it was about you.

*Who can see your data?*

However, some people can see your name and other personal information without a code. This can be data collected specifically for this study, but also data from your medical file.

These are people who check whether the researchers are conducting the research properly and reliably. These people can access your data:

- Members of the committee that monitors the safety of the investigation.
- A controller who works for the researcher.
- National and international supervisory authorities.

These people keep your data confidential. We ask you to give permission for these persons to inspect it. The Health and Youth Care Inspectorate can view your data without your permission.

*How long do we keep your data and body material?*

We keep your data in the hospital for 25 years. We also store your body material in the hospital. It will be kept for 5 years in order to be able to make new determinations related to this research in the course of this research. To enable possible follow-up research in other studies (see also the heading below), your body material will be stored for 20 years. As soon as this is no longer necessary, we destroy your body material.

*Can we use your data and body material for other research?*

Your collected data and your (remaining) body material may also be important for other scientific research in the field of (intolerance to) cholesterol-lowering drugs. For this purpose, your data and body material will be stored in the hospital. In the consent form, you indicate whether you agree to this. Do you not give permission? Then you can still participate in this study. You will receive the same care.

*Can you withdraw your consent to the use of your data?*

You can revoke your consent to the use of your data at any time. Then tell the researcher. This applies to the use in this study and to the use in other research. But beware: do you withdraw your consent, and have researchers already collected data for a study? Then they may still use this data. For your body material, the researchers will destroy it after you withdraw your consent. But have measurements already been taken with your body material? Then the researcher may continue to use the results.

*Want to know more about your privacy?*

- Would you like to know more about your rights regarding the processing of personal data? Then take a look at [www.autoriteitpersoonsgegevens.nl](http://www.autoriteitpersoonsgegevens.nl).
- Do you have questions about your rights? Or do you have a complaint about the processing of your personal data? Please contact the person responsible for processing your personal data. For your research, this is [LOCATION]. See Appendix A for contact details, and website.
- If you have any complaints about the processing of your personal data, we recommend that you first discuss them with the investigation team. You can also go to the Data Protection Officer of [LOCATION]. Or you can file a complaint with the Dutch Data Protection Authority.

*Where can you find more information about the study?*

You can find more information about the study on the following website(s): [www.clinicaltrialsregister.eu](file:///C:/Users/melvinlafeber/Library/CloudStorage/OneDrive-ErasmusMC/PCSK9i/verhofr/AppData/Local/Microsoft/Windows/INetCache/Application%20Data/Anouk/DCRF%20werkgroep%20PIF/www.clinicaltrialsregister.eu) and/or <https://euclinicaltrials.eu>. After the research, the website can show a summary of the results of this research. You can find the survey by searching for 'EU 2023-507489-20-00'

1. **Will you be compensated if you participate in the study?**

The research resources and extra tests for the research do not cost you anything extra. You will therefore not receive any compensation if you participate in this study. However, you will be reimbursed for any additional travel costs. This is a reimbursement of your travel costs per additional visit to the hospital if you are assigned to group 1 of the study, as you will have to make 4 additional visits to the hospital.

1. **Are you insured during the study?**

Insurance has been taken out for everyone who participates in this study. The insurance pays for damage caused by the study. But not for all damage. In **Appendix B** you will find more information about the insurance and the exceptions. It also states to whom you can report damage.

1. **We inform your general practitioner, treating specialist and your own pharmacy and request information from them.**

The researcher will send a letter and/or e-mail to your general practitioner, treating specialist and your own pharmacy to let them know that you are participating in the study. This is for your own safety, so that your own doctors and pharmacist are aware of the medicines we are administering to you in this study and so that appropriate care can be provided in the event of problems or complications. For the study, it is also important to receive information about diseases you are familiar with and your medication use at various times during the study. For this purpose, we also request information from your general practitioner, treating specialist and pharmacy during the study.

1. **Do you have any questions?**

Questions about the research can be asked to the research team. Do you want advice from someone who has no interest in it? Then go to the independent expert, for contact details see Appendix A. She knows a lot about the research, but does not cooperate with this research.

Do you have a complaint? Then discuss this with the researcher. Would you rather not do this? Then go to the complaints officer of your hospital or file a complaint with the Dutch Data Protection Authority. Appendix A shows where you can find them.

1. **How do you give permission for the study?**

You can first think calmly about this research. Then you tell the researcher whether you understand the information and whether or not you want to participate. Would you like to participate? Then you must fill in the consent form that you will find with this information letter in Appendix E. You and the researcher will both receive a signed version of this consent form.

Thank you for your time.

1. **Attachments to this information**

A. Contact details [LOCATION]

B. Insurance information

C. Schedule of research operations with description and overview of measurements

D. Package leaflet of rosuvastatin

E. Consent form for the subject

**Appendix A: contact details [LOCATION]**

On this page, study-site specific contact details are provided to the study participants.

**Appendix B: insurance information**

Erasmus MC has taken out insurance for everyone who participates in the study. The insurance pays for the damage you have because you participated in the study. This concerns damage that you receive during the study, or within 4 years after the end of your participation in the study. You must report damage to the insurer within 4 years.

Have you suffered damage as a result of the investigation? Then report this to this insurer:

The insurer of the study is:

Name of insurer: Centramed B.A.

Address: P.O. Box 7374

2701 AJ Zoetermeer

Telephone number: 0703017070

Email: schade@centramed.nl

Policy number: 624.100.042

The insurance pays a maximum of €650,000 per test subject with a maximum of €5,000,000 for the entire study and €7,500,000 per year for all studies from the same client.

Please note that the insurance does not cover the following damages:

- Damage due to a risk about which we have provided you with information in this letter. But this does not apply if the risk turned out to be greater than we thought beforehand. Or if the risk was very unlikely.
- Damage to your health that would have occurred even if you had not participated in the study.
- Damage caused by the fact that you did not follow directions or instructions or did not follow them properly.
- Damage to the health of your children or grandchildren.
- Damage due to a treatment method that already exists. Or by researching a treatment method that already exists.

These provisions are contained in the 'Decree on compulsory insurance for medical-scientific research involving human subjects 2015'. This decree is in the Government Laws Bank (<https://wetten.overheid.nl>).

**Appendix C: Schedule of research operations with description and overview of measurements**

| **Timing (+/- weeks)** | **First visit** | **Visit for group assignment**  **(0 weeks)** | **6 weeks**  **(+/- 1 week)** | **12 weeks (+/- 1 week)** | **18 weeks**  **(+/- 1 week)** | **24 weeks**  **(+/- 1 week)** | **6 months**  **(+/- 4 weeks)** | **9 months**  **(+/- 6 weeks)** | **12 months**  **(+/- 6 weeks)** | **15 months**  **(+/- 6 weeks)** | **18 months**  **(+/- 6 weeks)** | **21 months**  **(+/- 6 weeks)** | **24 months**  **(+/- 6 weeks)** | **36 months**  **(+/- 6 weeks)** | **48 months**  **(+/- 6 weeks)** |
| --- | --- | --- | --- | --- | --- | --- | --- | --- | --- | --- | --- | --- | --- | --- | --- |
| **Hospital visit?** | **Yes** | **Yes** | **Yes** | **Yes** | **Yes** | **Yes** | **Yes** | **No** | **Yes** | **No** | **No** | **No** | **Yes** | **Yes** | **Yes** |
| Discussion about the nocebo effect that can occur with statin use |  | 〇 |  |  |  |  | ∗ |  |  |  |  |  |  |  |  |
| Keeping track of side effects in Erasmus MC's DigiZorg phone app by means of questionnaires  *(3x/week and in the last week period 1x/day)* |  | ∗ | ∗ | ∗ | ∗ | ∗ |  |  |  |  |  |  |  |  |  |
| Keeping track of side effects in Erasmus MC's DigiZorg phone app by means of questionnaires  *(1x/week)* |  | 〇 | 〇 | 〇 | 〇 | 〇 |  |  |  |  |  |  |  |  |  |
| Measurements in the hospital |  | ⊛ |  |  |  |  | ⊛ |  | ⊛ |  |  |  | ⊛ | ⊛ | ⊛ |
| Blood sampling in the hospital |  | ⊛ | ∗ | ∗ | ∗ | ∗ | ⊛ |  | ⊛ |  |  |  | ⊛ | ⊛ | ⊛ |
| Questionnaires digital and other data collected digitally or in the hospital |  | ⊛ |  | ⊛ |  |  | ⊛ | ⊛ | ⊛ | ⊛ | ⊛ | ⊛ | ⊛ | ⊛ | ⊛ |

∗ = Research actions and measurements that are only performed in group 1

〇 = Research acts and measurements taken only in group 2

⊛ = Research actions and measurements taken in both groups

**Appendix D: Package leaflet of rosuvastatin**

You can find the package leaflet for rosuvastatin on the Medicines Information Bank website. The link to this is as follows: <https://www.geneesmiddeleninformatiebank.nl/bijsluiters/h26872.pdf>

**Appendix E: consent form for the subject**

Belonging to

*Side effects of statins due to negative expectations of the drug*

- I have read the information letter. I was also able to ask questions. My questions have been answered well enough. I had enough time to decide whether to participate.
- I know that participating is voluntary. I also know that I can decide at any time not to participate in the study. Or to stop it. I don't have to say why I want to stop.
- I give the researcher permission to let my general practitioner, treating specialist(s) and my own pharmacy know that I am participating in this study.
- I know that I will be enrolled for this study at Erasmus MC as a patient with the minimum number of data required.
- I give the researcher permission to request information from my general practitioner, pharmacy and/or treating specialist(s) about my medical history and about my medication use.
- I give the researchers permission to collect and use my data and body material. The researchers are only doing this to answer the research question of this study.
- I know that for the purpose of checking the research, some people can see all my data. Those people are listed in this information letter. I give these people permission to view my data for this check.
- I know that I am not allowed to become pregnant during the first 12 months of this study.
- The researcher discussed with me how best to prevent myself from becoming pregnant.
- Would you like to tick yes or no in the table below?

| I give permission to keep my data to use it for other research, as stated in the information letter. | Yes ☐ | No☐ |
| --- | --- | --- |
| I give permission to keep my (remaining) body material to use it for other research, as stated in the information letter. The body material is kept for another 20 years. | Yes ☐ | No☐ |
| I give permission to ask me if I want to participate in a follow-up study after this study. | Yes ☐ | No☐ |

- I want to participate in this research.

My name is (test subject): ......................................

Signature:........................... Date : __ / __ / __

-----------------------------------------------------------------------------------------------------------------

I declare that I have fully informed this subject about this study.

Is there any information that may influence the subject's consent during the study? Then I let this test subject know in time.

Name of investigator (or his representative):.....................................

Signature:........................... Date:__/__/__

-----------------------------------------------------------------------------------------------------------------

*The subject will receive a complete information letter, together with a signed version of the consent form.*
