# Supplementary material for: N=1-studies In Statin-intolerance; Objectifying Nocebo Effects (NISONE): a study protocol for a randomised controlled trial assessing the implementability of N=1-studies to promote the use of statins
Source: BMJ Open. 2026 Jun 25;16(6):e110978. doi: 10.1136/bmjopen-2025-110978 (PMC13311702; doi:10.1136/bmjopen-2025-110978)
Supplement: Supplementary file 2 [file bmjopen-2025-110978.reviewer_comments.pdf]

PEER REVIEW HISTORY

BMJ Open publishes all reviews undertaken for accepted manuscripts. Reviewers are asked to complete a checklist review form and are provided with free text boxes to elaborate on their assessment. These free text comments are reproduced below.

ARTICLE DETAILS

Title (Provisional)

N=1-studies In Statin-intolerance; Objectifying Nocebo Effects (NISONE): a study protocol for a randomized controlled trial assessing the implementability of N=1-studies to promote the use of statins

Authors

Mijnster, Ruben John Matthijs; Roeters van Lennep, Jeanine; Rietdijk, Wim J.r.; Akgöl, Kübra; Kemper, Elles Marleen; van den Berg, Daniëlle; van der Kuy, Hugo M.; Lafeber, Melvin

---

VERSION 1 - REVIEW

**Reviewer**	**1**
**Name**	**D'Ascenzo, Fabrizio**
**Affiliation**	**San Giovanni Battista, Molinette**
**Date**	**16-Jan-2026**
**COI**	**None**

---

all comments have beeen addressed

**Reviewer**	**2**
**Name**	**Obokata, Masaru**
**Affiliation**	**Gunma University**
**Date**	**12-Feb-2026**
**COI**	**None**

Mijnster and colleagues provide a protocol paper regarding a study protocol for a randomized controlled trial assessing the implementability of N=1-studies to promote the use of statins, which is unique and interesting. I have some comments and suggestions to improve the manuscript.

1. Please provide the rationale for the sample size calculation assuming a 12% continuing rate of statin in the control group and a 30% rate in the N-1-intervention group.

2. Please clarify the definition of “continuous statin use” at the one-year mark. Is it defined by adherence? For example, if a patient took statins for half the month, would that be defined as continuous use?

3. Please clarify how these interests of the secondary outcome are defined: quality of life; cost-effectiveness; views on medication; satisfaction with statins; trust in the HCP; and HCP satisfaction with intervention.

**Reviewer**	**3**
**Name**	**Kronish, Ian**
**Affiliation**	**Columbia University Medical Center**
**Date**	**11-Apr-2026**
**COI**	**None**

This manuscript describes the study protocol for a convention parallel group RCT comparing an N-of-1 strategy vs usual care for patients with perceived-statin associated myalgia symptoms and established CAD or familial hypercholesterolemia – patients groups who have clear benefits from statins. The rationale for the trial was well described. The study addresses an important research question regarding the possibility of using N-of-1 trials to overcome the presumed nocebo effect of statins. The use of conventional RCT design to study the clinical effectiveness of the N-of-1 strategy, while not new, is relatively innovative. The paper appropriately references and follows the SPIRIT guidelines. The inclusion of patients in the design of the protocol was a strength. Some comments that may be useful for improving the manuscript are provided below:

1. The study design section could explicitly explain the rationale for the 2:1 randomization

2. The study design could also explain the rationale for the N-of-1 sequences.

3. Details on the recruitment methods were not provided which would make it challenging to understand generalizability to other settings. It would also be helpful to track the proportion of patients approached who agree to participate.

4. The rationales for the statin type and dose and option for increasing dose were not provided.

5. Additional details on the counseling session at which patients have their N-of-1 data discussed could be provided as this is a key ingredient. How long? What format? What context – within an existing primary care visit, in-person? How will N-of-1 data be described/visualized for patients? Additional details can also be provided regarding counseling in the usual care group.

6. The rationale for measuring lipids at the end of each treatment period could be provided.

7. Additional details could be provided on the rationales for the key measures. Further, additional details should be provided on measures that do not cite an existing validated instrument such as the reasons for stopping medication and barriers to medication adherence.

8. The sample size estimate does not provide any citations to justify the inputs to its power calculations.

9. The statistical analysis plan did not provide many details, such as accounting for clustering by HCP and handling stratification in analyses. Similarly, scant details were provided for analysis of cost-effectiveness data.

10. The statistical analysis plan does not describe how the N-of-1 data will be aggregated or visualized to describe symptoms on and off statins.

VERSION 1 - AUTHOR RESPONSE

Reviewer 1

Dr. Fabrizio D'Ascenzo, San Giovanni Battista

Comments to the Author:

All comments have been addressed.

Reviewer 2

Dr. Masaru Obokata, Gunma University

Comments to the Author:

Mijnster and colleagues provide a protocol paper regarding a study protocol for a randomized controlled trial assessing the implementability of N=1-studies to promote the use of statins, which is unique and interesting. I have some comments and suggestions to improve the manuscript.

1. **Please provide the rationale for the sample size calculation assuming a 12% continuing rate of statin in the control group and a 30% rate in the N-1-intervention group.**

   We thank the reviewer for this valuable point of feedback. The reviewer is correct that it is important to provide a clear justification for the numbers used in a power analysis in a protocol manuscript.

   For this protocol, we assumed that 30% of patients in the intervention group would be willing to continue statin-therapy, which we considered a conservative estimate. Several

previous studies have investigated this outcome. Two of these studies (references: (1) *Joy TR, Monjed A, Zou GY, Hegele RA, McDonald CG, Mahon JL. N-of-1 (single-patient) trials for statin-related myalgia. Ann Intern Med. 2014;160(5):301-10* and; (2) *Tudor K, Brooks J, Howick J, Fox R, Aveyard P. Unblinded and Blinded N-of-1 Trials Versus Usual Care: A Randomized Controlled Trial to Increase Statin Uptake in Primary Care. Circ Cardiovasc Qual Outcomes. 2022;15(6):e007793*) reported the lowest lower bounds of the 95% confidence intervals, finding proportions of patients willing to continue statin use after 12 months of 71% (95% CI 29%–96%) and 46% (95% CI 29%–63%) respectively. To ensure a cautious estimate for our power calculation, we based our assumption on the lowest bound of these confidence intervals: approximately 30%.

With regard to the 12% in the usual care group, we believe the true proportion of patients willing to continue statin therapy in this group may in fact be lower (near 0%). However, we considered that some degree of contamination bias may occur, as physicians may apply insights gained from the intervention arm in routine care, for example through improved communication regarding statin intolerance and nocebo effects. This could lead to a higher proportion of patients in the usual care group choosing to continue treatment. Therefore, we selected an estimate of approximately 12% for this group. We have now better clarified this rationale in the manuscript:

Page 14 (lines 2-16): "A power analysis was performed to estimate the required number of participants for the study. This calculation assumed that 30% of patients will continue using statins after the N=1-intervention compared to 12% in the usual care group and included a drop-out margin of 25%. We have based the assumption that 30% of patients will continue statin therapy in the N=1-intervention arm of the study on four previous N=1-studies conducted in patients with statin-intolerance that assessed the proportion of patients who restarted or were willing to restart statin-therapy.[13, 14, 23, 24] Two of these studies reported the lowest lower bounds of the 95%-CIs for this outcome of 29%.[23, 24] To ensure a conservative estimate for our power calculation, we therefore based our assumption on this lower bound. We have also assumed that 12% of patients in the usual care arm will continue statin-therapy, which we consider a cautious estimate. However, we anticipate that some contamination bias may occur, as physicians may apply insights gained from the N=1-intervention arm in routine care, for example through improved communication regarding statin-intolerance and nocebo effects. This could lead to a higher proportion of patients in the usual care arm choosing to continue treatment."

2. **Please clarify the definition of "continuous statin use" at the one-year mark. Is it defined by adherence? For example, if a patient took statins for half the**

month, would that be defined as continuous use?

We thank the reviewer for this valuable point of feedback, as it is important to clearly specify how the primary outcome is defined. However, under the section "planned outcomes", we have already described the primary outcome measure as follows:

Page 12 (lines 24-25): "The primary outcome of the study is the incidence of self-reported statin-use at 12 months after randomization, …"

As this definition already implies, this is a binary outcome (yes/no: statin-use versus no statin-use). In addition, medication adherence is assessed separately using the 5-item Medication Adherence Reporting Scale (MARS-5), which is a secondary outcome as specified in the same paragraph.

To better clarify the solely binary aspect of our primary outcome, we have now clarified this in the manuscript:

Page 12 (lines 24-25): "The primary outcome of the study is the incidence of self-reported statin-use at 12 months after randomization, which is a binary outcome measure (yes/no)."

Please clarify how these interests of the secondary outcome are defined: quality of life; cost-effectiveness; views on medication; satisfaction with statins; trust in the HCP; and HCP satisfaction with intervention.

We thank the reviewer for this valuable point of feedback. We agree that we should have more clearly specified how these outcome measures are assessed. Most of the secondary outcome measures are collected using questionnaires, as summarized in the section "Measurements and data collection". For the outcomes mentioned by the reviewer, as well as other secondary outcomes, we have now clarified in the manuscript which specific questionnaires are used to define each outcome:

Page 13, lines (9-23): "All secondary outcomes that are studied are:

- statin-use;
- PCSK9i-use;
- LDL-C levels;
- quality of life (EQ-5D-5L questionnaire);
- reported symptoms;

- cost-effectiveness (iMCQ- and iPCQ-questionnaire, among others);
- views on medication (BMQ-questionnaire);
- satisfaction with statins (PESaM-questionnaire);
- decisional conflict (DCS-questionnaire);
- trust in the HCP (WFPTS-questionnaire);
- HCP satisfaction with intervention (self-made, non-validated questionnaire with statements about acceptability on a 5-point Likert-scale; disagree-agree);
- treatment concordance during intervention;
- physical and mental complaints during intervention."

Reviewer 3

Dr. Ian Kronish, Columbia University Medical Center

Comments to the Author:

This manuscript describes the study protocol for a convention parallel group RCT comparing an N-of-1 strategy vs usual care for patients with perceived-statin associated myalgia symptoms and established CAD or familial hypercholesterolemia – patients groups who have clear benefits from statins. The rationale for the trial was well described. The study addresses an important research question regarding the possibility of using N-of-1 trials to overcome the presumed nocebo effect of statins. The use of conventional RCT design to study the clinical effectiveness of the N-of-1 strategy, while not new, is relatively innovative. The paper appropriately references and follows the SPIRIT guidelines. The inclusion of patients in the design of the protocol was a strength. Some comments that may be useful for improving the manuscript are provided below:

1. **The study design section could explicitly explain the rationale for the 2:1 randomization**

   We thank the reviewer for this valuable suggestion. The reviewer is right that the rationale to choose a 2:1 randomization ratio remains a bit unclear now in the manuscript. We have now better clarified the rationale behind this strategy in the manuscript:

   Page 7 (lines10-15): "A 2:1 randomization ratio is selected because it is advantageous for the power calculation, minimizing the number of participants required for the study. In addition, it ensures that the majority of participants receive the N=1 intervention, allowing for better-powered analyses for outcomes in which

patients identified through the intervention as intolerant due to statin-therapy or intolerant due to misattribution and/or nocebo effects are compared to each other."

2. **The study design could also explain the rationale for the N-of-1 sequences.**
   We thank the reviewer for this valuable point of feedback. In the "study design" section of the manuscript, we had briefly described the rationale for the three treatment sequences in the N=1-intervention arm. However, we agree that this could be further clarified. Therefore, we have revised this section to provide a clearer explanation as follows:

   Pages 7 (lines 21-26) and 8 (line 1): "Possible treatment sequences for the N=1-intervention are designed in a way to ensure a balanced exposure to statin and placebo treatment across the first four periods of the intervention (i.e., two statin periods and two placebo periods). Also a treatment sequence with two consecutive periods of placebo treatment is excluded to avoid a prolonged use of placebo treatment. Consequently, in the intervention-arm of the study, participants will undergo an N=1-intervention according to three different possible treatment sequences which are double-blinded (**Table 1**)."

3. **Details on the recruitment methods were not provided which would make it challenging to understand generalizability to other settings. It would also be helpful to track the proportion of patients approached who agree to participate.**
   We thank the reviewer for this valuable suggestion. The reviewer is correct that our manuscript did not adequately address the important aspect of patient recruitment. We agree that this should be more clearly described. Consequently, we have now added a separate section, "Recruitment of patients", to the manuscript. In this section, we describe the recruitment strategy, as well as the procedure for documenting the proportion of patients willing to participate and the reasons for declining participation:

   Page 11 (lines 8-18):
   "**Recruitment of patients**
   Patients will be recruited via the outpatient clinics of all participating centers in this study. Recruitment will take place through cardiology and vascular medicine clinics in regional hospitals and academic medical centers, as these specialists are the only healthcare professionals authorized to prescribe PCSK9i to adults in the Netherlands. Potential participants will be asked whether they are interested in participating in the trial after receiving information about the study from their treating healthcare professional. They will be given at least one week to consider whether or

not they wish to participate. With the consent of potential participants, reasons for non-participation will be recorded in order to assess both the proportion of eligible patients who agree to participate and the reasons for declining participation."

To avoid redundancy in the manuscript after this addition, we have deleted the following part of the manuscript:

Page 10 (lines 18-19): "Participants will be recruited from clinics of all participating hospitals."

4. **The rationales for the statin type and dose and option for increasing dose were not provided.**

   We thank the reviewer for this valuable point of feedback. We agree that the rationale should be more clearly elaborated in the manuscript. We specifically selected rosuvastatin and atorvastatin as the statins of choice because these agents have the greatest potency for lowering LDL-cholesterol levels. As a result, even at relatively low doses, guideline-recommended LDL-cholesterol targets can more readily be achieved.

   Furthermore, we chose a flexible dosing regimen of either a single or double daily dose of these statins (rosuvastatin 10 mg 1–2 tablets/day or atorvastatin 20 mg 1–2 tablets/day), to allow the treating healthcare professional to individualize dosing and optimize the likelihood of achieving guideline-recommended LDL-cholesterol targets. We have now clarified this rationale in the manuscript as follows:

   Page 8 (lines 4-9): "Rosuvastatin and atorvastatin were chosen as the statins of choice due to their relatively high LDL-C lowering potency as compared to other statins. We specifically included the option of either a single daily dose of rosuvastatin 10 mg or atorvastatin 20 mg, or administering a double dose, to allow the treating healthcare professional to individualize dosing and thereby optimize the likelihood of achieving guideline-recommended LDL-C targets."

5. **Additional details on the counseling session at which patients have their N-of-1 data discussed could be provided as this is a key ingredient. How long? What format? What context – within an existing primary care visit, in-person? How will N-of-1 data be described/visualized for patients? Additional details can also be provided regarding counseling in the usual care group.**

We thank the reviewer for this valuable point of feedback. In the revised version of the manuscript, we have now more extensively elaborated on the counselling regarding the results of the N=1-intervention and how this is conducted.

For the usual care group, we have deliberately not provided detailed guidance on this aspect, as there is currently no specific guideline or standardized approach describing how discussions about statin-intolerance and its potential causes should be conducted in routine clinical practice. Therefore, to avoid over-standardization and to better reflect real-world practice, we have left this largely to the discretion of the treating healthcare professionals with only limited guidance, which we believe best reflects the usual care at this moment.

Pages 8 (lines 23-27) and 9 (lines 1-7): "During this period, participants will receive counseling on their individually reported symptoms and cholesterol levels during the treatment periods. They will receive a generated report consisting of several figures and graphs, which will among other things show the number of (muscle) symptoms during statin versus placebo periods, cholesterol levels across the different periods, the distribution of symptoms across different body regions and the extent to which symptoms affected their daily functioning, including any differences between statin and placebo periods. In addition, participants will receive a video that further explains their personal results. Subsequently, they will discuss their individual results in a consultation of approximately 30 minutes with their treating healthcare professional. During this consultation, the results will be reviewed in detail and further interpretation will be provided, including whether the findings are suggestive of statin-attributable symptoms or symptom misattribution and nocebo effects."

6. **The rationale for measuring lipids at the end of each treatment period could be provided.**

   We thank the reviewer for this valuable point of feedback. We agree with the reviewer that it is important to elaborate on this study design choice. We have built in the measurement of lipid levels after the first four treatment periods for two main reasons: firstly to provide participants with an insight in what their lipid levels are under statin- and placebo-therapy (which might encourage statin-use) and secondly to allow verification that the correct treatment was taken during each period. We have now further clarified this in our manuscript:

   Page 8 (lines 12-19): "Furthermore, in the sixth week of every treatment period, participants will have their blood drawn for cholesterol levels. Cholesterol levels during these treatment periods are measured to provide participants with insight into

their lipid levels under both statin- and placebo- therapy. This may help motivate patients to continue statin-use and also allows verification that the correct treatment was taken during each period. This is important to address potential concerns from participants that treatment periods may have been inadvertently mixed up or that they may have incorrectly taken the assigned medication, which could otherwise lead to doubts about the validity of their individual results."

7. **Additional details could be provided on the rationales for the key measures. Further, additional details should be provided on measures that do not cite an existing validated instrument such as the reasons for stopping medication and barriers to medication adherence.**
We thank the reviewer for this important point of feedback. However, we do want to note to the reviewer that there is only one 'key measure' (primary outcome) in this study, which is the statin-use of participants at the timepoint of 12 months. We have now further elaborated on why we feel this outcome should be the primary outcome of the study in the manuscript:

Pages 12 (lines 25-26) and 13 (lines1-5): "We specifically chose this outcome as the primary endpoint because the main objective of the study is to determine whether N=1-interventions, compared with usual care, can increase statin-use in patients in routine clinical practice. We considered the 12-month timepoint to be the most appropriate to evaluate this outcome, as we believe this timepoint, approximately six months after completion of the N=1-intervention, best reflects patients' willingness to continue statin-therapy in the longer term, beyond the immediate influence of the intervention."

Furthermore, the reviewer correctly addresses that additional details should be provided on the measures that are mentioned in the manuscript, that do not cite a validated instrument. Data collection about barriers to medication adherence, reasons for stopping medication and changes in lifestyle are done in a questionnaire that only contains an open-ended text-field querying about these topics. Participant acceptability of the intervention is queried using a self-made, non-validated questionnaire We have now elaborated on this a bit more in the manuscript:

Page 12 (lines 4-21): "All items that these questionnaires consist of are:

- demographics;
- self-reported medication use;

- barriers to medication adherence (open-ended question);
- reasons for stopping medication (open-ended question);
- changes in lifestyle (open-ended question);
- participant acceptability of N=1-intervention (self-made, non-validated questionnaire with statements about acceptability on a 5-point Likert-scale; disagree-agree);
- 5-item Medication Adherence Reporting Scale (MARS-5);
- EuroQoL-5 Dimensions-5 Levels (EQ-5D-5L);
- Set of Brief Screening Questions (SBSQ);
- Beliefs about Medicines Questionnaire (BMQ);
- Patient Experiences and Satisfaction with Medication (PESaM);
- Decisional Conflict Scale (DCS);
- Wake Forest Physician Trust Scale (WFPTS);
- Psychological Inventory of Financial Scarcity on 4 items (PIFS-4);
- iMTA Medical Consumption Questionnaire (iMCQ);
- iMTA Productivity Costs Questionnaire (iPCQ).

8. **The sample size estimate does not provide any citations to justify the inputs to its power calculations.**
   We thank the reviewer for this great point of feedback. We have received the same feedback from reviewer 2. In line with his feedback, we have now better clarified the inputs to the power calculations in our revised manuscript:

   Page 14 (lines 2-16): "A power analysis was performed to estimate the required number of participants for the study. This calculation assumed that 30% of patients will continue using statins after the N=1-intervention compared to 12% in the usual care group and included a drop-out margin of 25%. We have based the assumption that 30% of patients will continue statin therapy in the N=1-intervention arm of the study on four previous N=1-studies conducted in patients with statin-intolerance that assessed the proportion of patients who restarted or were willing to restart statin-therapy.[13, 14, 23, 24] Two of these studies reported the lowest lower bounds of the 95%-CIs for this outcome of 29%.[23, 24] To ensure a conservative estimate for our power calculation, we therefore based our assumption on this lower bound. We have also assumed that 12% of patients in the usual care arm will continue statin-therapy, which we consider a cautious estimate. However, we anticipate that some contamination bias may occur, as physicians may apply insights gained from the N=1-intervention arm in routine care, for example through improved communication regarding statin-intolerance and nocebo effects. This could lead to a

higher proportion of patients in the usual care arm choosing to continue treatment.”

9. **The statistical analysis plan did not provide many details, such as accounting for clustering by HCP and handling stratification in analyses. Similarly, scant details were provided for analysis of cost-effectiveness data.**
   We thank the reviewer for this important comment. We fully agree that the statistical analysis plan should ideally include more detailed specifications, such as accounting for clustering by HCP and the handling of stratification variables, as well as more detailed descriptions of the cost-effectiveness analyses. We acknowledge that these aspects are described only at a relatively global level in the current protocol manuscript. However, this reflects the level of detail as it is also specified in the underlying study protocol. As this manuscript is a protocol paper and is intended to remain consistent with the approved and implemented study protocol, it would not be appropriate to introduce additional analytical specifications at this stage that are not part of the original protocol. We feel this would not be scientifically sound and it would also not be in accordance with SPIRIT-guidelines.

   Therefore, no changes have been made to the manuscript in response to this comment.

9. **The statistical analysis plan does not describe how the N-of-1 data will be aggregated or visualized to describe symptoms on and off statins.**

We thank the reviewer for this valuable feedback. However, our response to this point is similar to that provided for comment 9. In the underlying study protocol, the methods for aggregating and visualizing the N=1-data are not specified in detail. As this manuscript is a protocol paper and is intended to remain fully consistent with the approved study protocol, it would not be appropriate to introduce additional analytical or methodological specifications at this stage that are not part of the original protocol. We consider it important from a scientific perspective to remain faithful to the predefined protocol without post-hoc additions, which would also not be in accordance with SPIRIT-guidelines.

Therefore, no changes have been made to the manuscript in response to this comment

---

VERSION 2 - REVIEW

**Reviewer**	**3**
**Name**	**Kronish, Ian**
**Affiliation**	**Columbia University Medical Center**
**Date**	**27-Apr-2026**

COI

The authors provided thorough responses to the prior comments. My only remaining comment refers to the revision related to the rationale for using 2:1 randomization. I disagree with the comment that suggest this approach is more efficient for powering teh study statistically speaking. The other rationale provided was more reasonable.

VERSION 2 - AUTHOR RESPONSE

Thank you for reviewing our manuscript entitled: "N=1-studies In Statin-intolerance; Objectifying Nocebo Effects (NISONE): a study protocol for a randomized controlled trial assessing the implementability of N=1-studies to promote the use of statins" and for your interest in our study. We appreciate your feedback and the opportunity to resubmit a revised manuscript.

We have made changes in our manuscript in response to the feedback. Please find below our specific responses to the questions and comments provided. Corresponding changes are indicated in red font in a tracked and changed version of the revised manuscript and in this response letter.
